# Medically unexplained somatic symptoms and its association with functionality and childhood trauma in type-1 bipolar disorder

**DOI:** 10.1192/j.eurpsy.2022.915

**Published:** 2022-09-01

**Authors:** N.G. Usta Saglam, B. Ayık, A. Baş, F. Izci

**Affiliations:** Istanbul Erenkoy Training and Research Hospital for Psychiatry and Neurological Diseases, Psychiatry, Istanbul, Turkey

**Keywords:** somatic symptoms, Childhood Trauma, bipolar disorder, functionality

## Abstract

**Introduction:**

Somatic symptoms with the heterogeneous character that are not fully explained by a medical condition are common in bipolar disorder (BD) which might interfere with the choice of treatment, health care utilization, medical costs as well as functionality.

**Objectives:**

The purpose of this study was to evaluate somatic symptoms in remitted type 1 BD and to examine the association of somatization, functionality, and childhood trauma which is a known mediator of adult somatization.

**Methods:**

After excluding patients with medical comorbidities, 61 patients diagnosed with BD type-1 according to the Diagnostic and Statistical Manual of Mental Disorders-V (DSM-V) participated in the study. We required at least 8 weeks of remission and confirm it with Hamilton Depression Rating Scale (HDRS) and Young Mania Rating Scale (YMRS). Somatization Scale, Functioning Assessment Short Test (FAST) and Childhood Trauma Questionnaire (CTQ) were administered to the participants.

**Results:**

Somatization scores were significantly correlated with CTQ (r=.310, p=.016), FAST- total (r=.307, p=.016), FAST-financial issues (r=.357, p=.005) and FAST-interpersonal relationships (r=.320, p=.012) subscale scores while inversely correlated with years in education (r=-,305, p=.017). When a partial correlation was run to determine the relationship between somatization and functioning whilst controlling for childhood trauma, there was no statistically significant correlation between somatization and functioning (p=.076).

**Conclusions:**

Our study suggests childhood trauma may have a major influence on the relation between somatization and functionality in patients with type- 1 BD. When addressing physical symptoms in patients with type-1 BD, an integrated approach including childhood trauma should be considered.

**Disclosure:**

No significant relationships.

